# Model predictions of features in microsaccade-related neural responses in a feedforward network with short-term synaptic depression

**DOI:** 10.1038/srep20888

**Published:** 2016-02-08

**Authors:** Jian-Fang Zhou, Wu-Jie Yuan, Zhao Zhou, Changsong Zhou

**Affiliations:** 1 College of Physics and Electronic Information, Huaibei Normal University, Huaibei 235000, China; 2 Department of Physics, Centre for Nonlinear Studies and the Beijing-Hong Kong-Singapore Joint Centre for Nonlinear and Complex Systems (Hong Kong), Institute of Computational and Theoretical Studies, Hong Kong Baptist University, Kowloon Tong, Hong Kong; 3 Beijing Computational Science Research Center, Beijing 100084, China; 4 Research Centre, HKBU Institute of Research and Continuing Education, Virtual University Park Building, South Area Hi-tech Industrial Park, Shenzhen, China

## Abstract

Recently, the significant microsaccade-induced neural responses have been extensively observed in experiments. To explore the underlying mechanisms of the observed neural responses, a feedforward network model with short-term synaptic depression has been proposed [Yuan, W.-J., Dimigen, O., Sommer, W. and Zhou, C. Front. Comput. Neurosci. 7, 47 (2013)]. The depression model not only gave an explanation for microsaccades in counteracting visual fading, but also successfully reproduced several microsaccade-related features in experimental findings. These results strongly suggest that, the depression model is very useful to investigate microsaccade-related neural responses. In this paper, by using the model, we extensively study and predict the dependance of microsaccade-related neural responses on several key parameters, which could be tuned in experiments. Particularly, we provide a significant prediction that microsaccade-related neural response also complies with the property “sharper is better” observed in many contexts in neuroscience. Importantly, the property exhibits a power-law relationship between the width of input signal and the responsive effectiveness, which is robust against many parameters in the model. By using mean field theory, we analytically investigate the robust power-law property. Our predictions would give theoretical guidance for further experimental investigations of the functional role of microsaccades in visual information processing.

Microsaccades are the involuntary, fast, and very small eye movements that occur during visual fixation. Over the past decade, the behavioral properties and functional role of microsaccades have been widely investigated^1^,^2^,^3^,^4^,^5^,^6^,^7^,^8^,^9^,^10^,^11^,^12^,^13^. It has been found that microsaccades play an important functional role in counteracting visual fading. In order to study neural dynamical mechanism of microsaccades for counteracting perceptual fading, neural responses correlated with microsaccades have been extensively studied in experiments at different levels—from neuronal activities^7^,^11^,^14^,^15^ to electroencephalogram (EEG)^9^,^16^ and functional magnetic resonance imaging (fMRI)^5^,^12^—in a number of cortical areas involved in visual information processing, including V1^5^,^7^,^12^, V2^5^,^12^,^14^, V3^12^, V4^14^, and MT^12^,^15^.

Recently, a report gave an explanation for microsaccades in counteracting visual fading by constructing a feedforward network model with short-term depression (STD) at thalamocortical synapses^17^, alternative to the assumption of retinal adaptation. The adapted synapses subjected to STD led to response depression in V1, which induces visual fading because of sustained depression during fixation. Therefore, it is possible that the generation of microsaccades serves to counteract the STD-induced depression of neuronal activity in order to counteract visual fading. In particular, the depressed model successfully reproduced several microsaccade-related experimental observations. For example, the neural response after microsaccade is stronger when a rhythmically flashing stimulus bar is on during fixation, as compared to a condition in which the bar is always on (stationary, i.e., non-flashing). The response peak induced by microsaccade increases with the increasing of microsaccadic magnitude or velocity. Moreover, the increasing response reaches saturation for large microsaccadic magnitude or velocity.

It was found by model simulations, that the above results are attributed to the sensitivity of STD to the change of stimuli^17^. These findings strongly suggest that the depression model is very useful to investigate microsaccade-related neural responses. Indeed, computational studies have explored the effect of STD on network dynamics and found various rich dynamical behaviors^18^,^19^,^20^,^21^, suggesting many important roles of STD in neural computations. Thus, the depression model can be expected to produce other abundant features in microsaccade-related neural responses. Model predictions of such features would provide theoretical guidance for experimental investigations. In this paper, by using the model, we extensively study the features of microsaccade-related neural responses with respect to several key parameters, which could be experimentally tested in future.

## Methods

**Figure 1:**
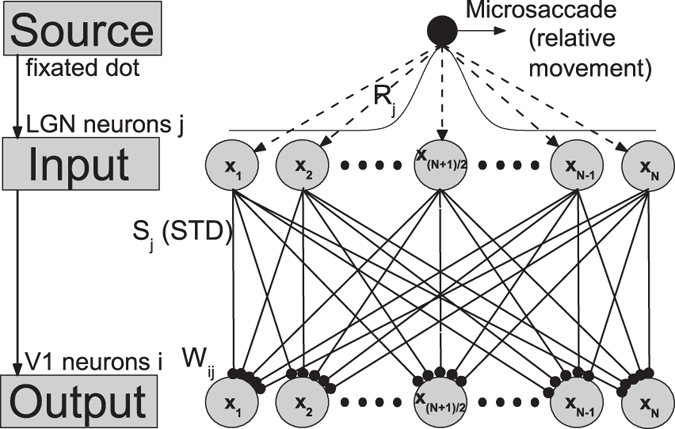
(Adapted from Ref. 17) The feedforward network model including STD during fixation with microsaccade. Here, neurons in LGN and V1 are labeled and arranged by the center positions *x*_*j*_ and 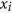 of their receptive fields in the ranges from −*L* to *L*, respectively. Gaussian filters (receptive fields) in LGN layer transform the afferent stimuli evoked by fixated dot into the inputs with Gaussian firing rate profile: 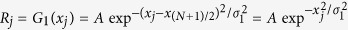. The *A* represents the amplitude of a visual input at fixated-dot position 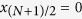. The *σ*_1_ is width of the tuning curve. The output layer V1 is connected to input layer LGN by thalamocortical synapses with synaptic strengths *S*_*j*_, which are subjected to the modification: STD. These connecting weights *W*_*ij*_ follow the Gaussian tuning curve: 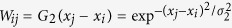, where 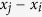 denotes denotes the position difference of receptive field centers between the input neuron *j* and the output neuron *i*. The microsaccade during fixation can be regarded as relative movement of the fixated dot over LGN with microsaccadic magnitude Δ*M*. In order to eliminate the effect of boundary owing to the limited scale of network, the corresponding input tuning curve *G*_1_ is extended to a period function with period 2*L*.

According to visual pathway^22^,^23^, a feedforward network model consisting of two layers corresponding to LGN and V1 with STD at thalamocortical synapses was proposed in Fig. 1^17^. Evoked by fixated dot, the LGN neuron *j* fires with rate *R*_*j*_ following Gaussian tuning curve *G*_1_ (shown in Fig. 1). Then, the firings *R*_*j*_ are straightly projected to V1 neuron *i* by STD synapses with linking weights *W*_*ij*_ following Gaussian tuning curve *G*_2_ (shown in Fig. 1). The membrane potential *V*_*i*_ of V1 neuron *i* in output layer is described by


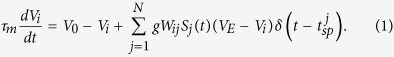


Here, we adopt the experimentally fitted parameter values 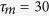 ms, *V*_0_ = −70 mV and *V*_*E*_ = 0 mV^24^,^25^. Each V1 neuron *i* integrates inputs coming from LGN neurons *j* at spike time 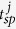 distributed as Poisson spike trains *R*_*j*_ by chemical couplings of *δ* function. When *V*_*i*_ reaches the threshold value −55 mV, neuron *i* emits a spike, and then *V*_*i*_ is reset to −58 mV. The parameter *g* denotes the maximal synaptic conductance. The synaptic strength *S*_*j*_(*t*) at thalamocortical system is subjected to the STD mechanism,


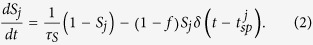


The parameter *f* (0.0 < *f* < 1.0) determines the amount of depression at synapse *j* induced by each spike in neuron *j*. The parameter 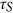 denotes the depression recovery time. When the afferent neuron *j* fires a Poisson spike train at rate *R*_*j*_, the synaptic strength will quickly decrease to the approximate steady state (SS, for a high rate *R*_*j*_)^24^,


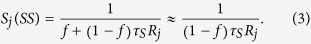


But, for a small firing rate *R*_*j*_, the synaptic strength approximately maintains its original value 1. This depression model gives a good fit of experimental data^24^. In the following simulations, we take *f* = 0.75 and 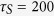 ms (except for parameter values in Fig. 8(a,b) for comparison), which lie within the range indicated in the experimental data^26^,^27^. The main qualitative results do not depend on the two parameters.

Experimentally, microsaccades are very fast movements^3^. In Ref. 17, we have studied the effect of finite microsaccadic speed and compared to experimental observations^9^. For simplicity, we here ignore the time course of microsaccades. In our simulations, microsaccade is regarded as a relative displacement ΔΜ of the tuning curve G1, which happens immediately. *N* neurons in LGN and V1 are spread uniformly in the ranges from −*L* to *L* (shown in Fig. 1), respectively. We count the total number of spikes 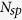 of the V1 neurons in a moving time bin *T* as a measure of the neural response. *N* = 1000 (except for parameter values in Fig. 8(d) for comparison), *L* = 10 and *T* = 50 ms (except for parameter values in Fig. 8(e) for comparison) are given. Choosing different parameter values *N*, *L* and *T*, however, does not alter the qualitative results.

## Results

In this section, our model predicts several new features of microsaccade-related neural responses which are likely testable in experiments. We first describe the dependence of microsaccade-induced neural responses on stimulus brightness, represented by the input amplitude *A*. Then, we study the effect of widths *σ*_1_ and *σ*_2_ of the two tuning curves *G*_1_ and *G*_2_. Finally, we provide a significant prediction that microsaccade-related neural response complies with a property “sharper is better”, which has been observed in many contexts in neuroscience, including orientation selectivity^28^, perceptual learning^29^,^30^ and auditory processing^31^. Interestingly, the property exhibits a robust power-law relationship between effectiveness of microsaccades and the width of Gaussian tuning curve *G*_1_.

### Dependence on stimulus brightness

**Figure 2:**
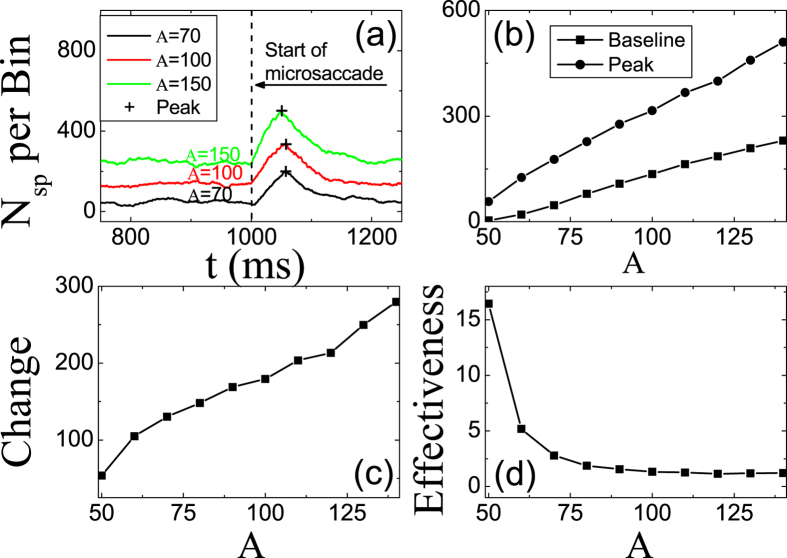
(**a**) Microsaccade-related neural activity for different brightness *A*. Microsaccade-related response baseline and peak (**b**), change (**c**) and effectiveness (**d**) for different brightness *A*. The parameters are *g* = 0.15, Δ*M* = 0.8 and *σ*_1_ = *σ*_2_ = 1.5. Data are averaged over 20 independent runs.

As shown in Eq. (3), the depression of synapse depends on the firing rate of the presynaptic LGN neuron. The higher the firing rate, the smaller the steady strength of the synapses. The stimulus brightness of fixation dot is denoted by amplitude *A* of the LGN neuron firing rate *R*_*j*_ in our model (shown in Fig. 1). Therefore, the stimulus brightness is expected to impact strongly on the microsaccade-related responses. Here, we simulated the effect of the amplitude *A* on neural responses induced by microsaccades. As shown in Fig. 2(a), a response peak appears soon after microsaccade, and the value depends strongly on *A*. In detail, we measure the dependance of baseline of neural activity before microsaccade, peak of neural activity after microsaccade, change as the difference between peak and baseline, and effectiveness as the ratio of change to baseline. Clearly, both the response baseline and peak increase linearly with the increasing of *A* when clear response can be induced for *A* > 50 (Fig. 2(b)) because the input 
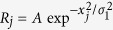
 from LGN neuron is proportional to *A*. We can write the linear relations of 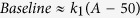 and 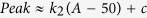, respectively. Here *k*_1_ and *k*_2_ denote the slopes of the two linear relations, and c is the value of *peak* when *A* is 50. Since the peak is always larger than the baseline, there is *k*_2_ > *k*_1_ > 0. So, the change 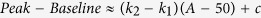 increases roughly linearly as the increasing of *A* (Fig. 2(c)). But, the effectiveness 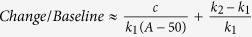 decreases as *A* becomes larger (Fig. 2(d)).

**Figure 3:**
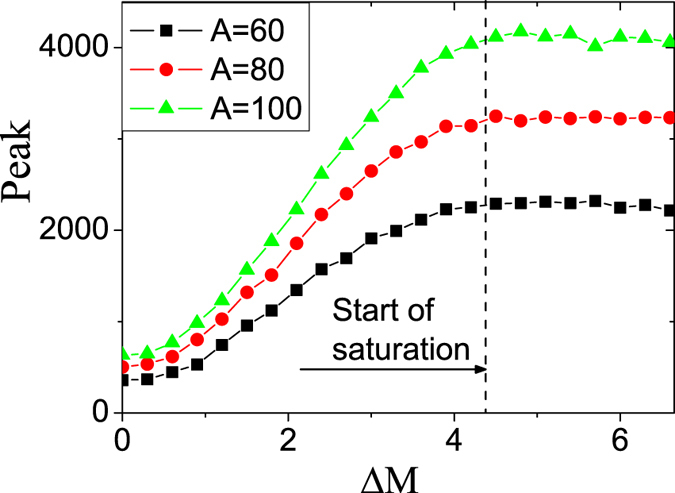
Saturation of the response peak for large microsaccades for different *A*. Dashed line denotes the similar threshold of microsaccade magnitude producing saturation for different *A*. Here *g* = 0.2, *σ*_2_ = 1.5 and *σ*_1_ = 1.5. Data are averaged over 20 independent runs.

Next, we studied effect of parameter *A* on the saturation of microsaccade-induced neural activity. In EEG experiment data, it has been recently found that^9^, neural response related with microsaccade increases with the increasing of microsaccade magnitude within the small region. The increasing response reaches a saturation value for larger microsaccade magnitudes. In our simulations, the response peak increases as the microsaccade magnitude ΔΜ with small size in Fig. 3. When the microsaccade magnitude increases to a threshold, the increasing response peak reaches to saturation, consistent with the experimental results in ref. 9. This saturation can be well explained as follows in our model^17^. When the moving distance due to large microsaccade exceeds the region with strong synapse-depression, the synaptic input will increase to the largest value (S*j* = 1) and become independent of the microsaccade magnitude, leading to saturated response. Here, we focus on effect of parameter *A* on the saturation. It is found that in Fig. 3, the saturation value becomes larger and larger with the increasing of *A*. It is because *A* denotes the stimulus strength of fixated dot. The large *A* corresponds to the large neural response 
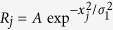
 in LGN, and so the large neural response and the responsive saturation value are produced in V1. We note that the threshold of microsaccade magnitude producing saturation is independent of *A*, since size of the synaptic depression region due to fixation is determined by the width of tuning curve *G*_1_ of firing *R*_*j*_, which is fixed in these simulations.

### Effect of width of tuning curves

**Figure 4:**
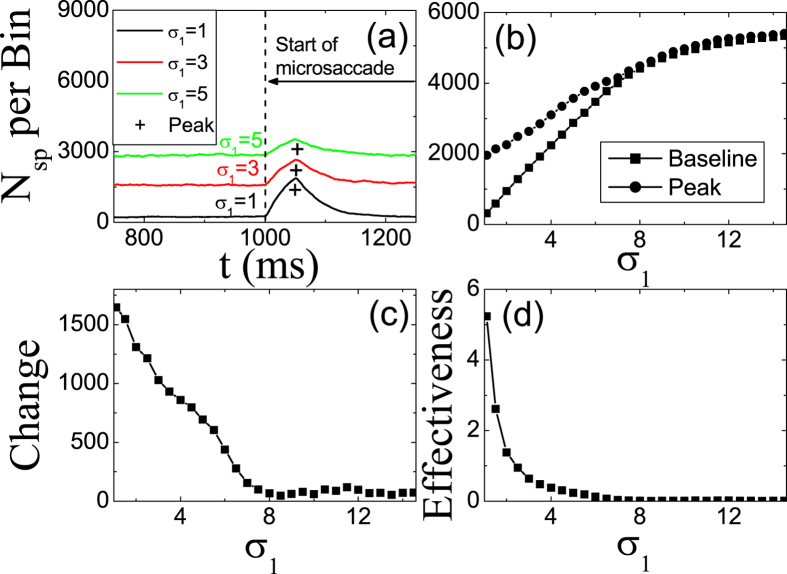
The same as in Fig. 2, but for different *σ*_1_. The parameters are *g* = 0.2, *A* = 100, Δ*M* = 2.0 and *σ*_2_ = 1.5.

We simulate the effect of the width *σ*_1_ of tuning curve *R*_*j*_ on microsaccade-induced neural responses in Fig. 4. It is found that, both of the response baseline and peak increase with the increasing of *σ*_1_ within the small region (Fig. 4(b)), because the larger *σ*_1_ denotes the larger inputs from LGN neurons due to the broader firing region of *R*_*j*_. When the *σ*_1_ increases to a certain value, the microsaccade with the fixed magnitude ΔΜ cannot move the fixated dot out of the depressed region of the synapses. The high response peak compared to baseline after microsaccade does not appear for the large *σ*_1_, i.e. the response peak tends to the response baseline (Fig. 4(b)). So, the change 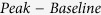 decreases approximately to zero with the increasing of *σ*_1_ (Fig. 4(c)). The decreasing *Change* and the increasing *Baseline* lead to the decreasing 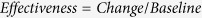 (Fig. 4(d)).

**Figure 5:**
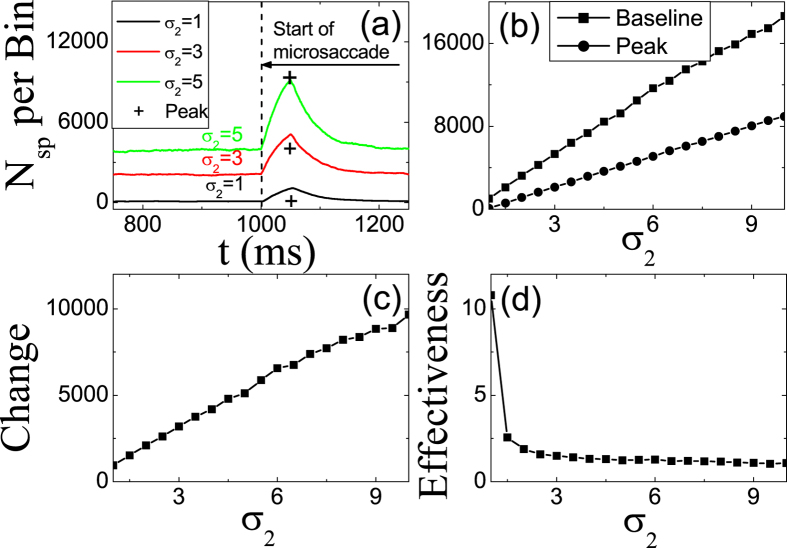
The same as in Fig. 2, but for different *σ*_2_. The parameters are *g* = 0.2, *A* = 100, Δ*M* = 2.0 and *σ*_1_ = 1.5.

Meanwhile, we simulate the effect of the width *σ*_2_ of tuning curve *W*_*ij*_ on microsaccade-induced neural responses in Fig. 5. Since the larger *σ*_2_ reflects the larger inputs from LGN neurons due to the broader coupling region of *W*_*ij*_, the response baseline and peak in V1 increase linearly as the increasing of *σ*_2_ (Fig. 5(b)). Similar to the effect of *A* in Fig. 2, the linear increasings of *Baseline* and *Peak* lead to the increasing of *Change* (Fig. 5(c)) and the decreasing of *Effectiveness* (Fig. 5(d)).

**Figure 6:**
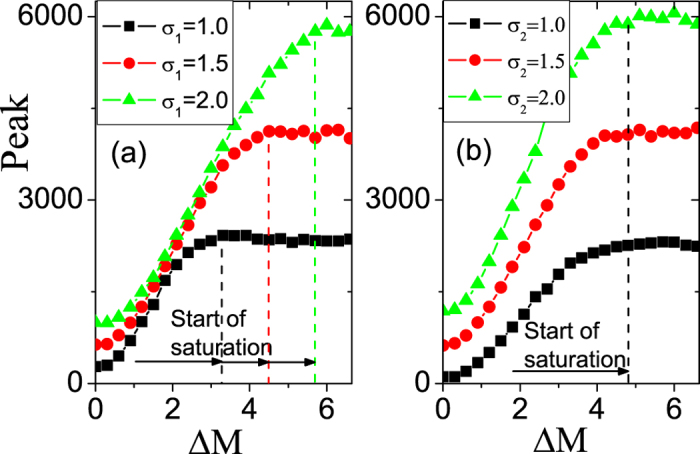
The same as in Fig. 3, but for different *σ*_1_ (**a**) and *σ*_2_ (**b**). Dashed lines denote the different thresholds of microsaccade magnitude producing saturation for different *σ*_1_ and *σ*_2_. Here *g* = 0.2, *A* = 100, *σ*_2_ = 1.5 (**a**) and *σ*_1_ = 1.5 (**b**).

Next, we study effects of parameters *σ*_1_ and *σ*_2_ on the saturation of microsaccade-induced neural activity with respect to size ΔΜ. As shown in Fig. 6, the saturation value becomes larger and larger with increasing *σ*_1_ or *σ*_2_. Since the *σ*_1_ denotes the response region in LGN and the *σ*_2_ reflects the coupling region from LGN to V1, larger neural response and the responsive saturation value are produced in V1 for larger *σ*_1_ or *σ*_2_. Particularly, a threshold of microsaccade magnitude producing the saturation depends strongly on *σ*_1_ (Fig. 6(a)), but not much on *σ*_2_ (Fig. 6(b)). With the smaller width *σ*_1_, the smaller microsaccade magnitude is required for producing a saturated response. Here, we can give the following understanding. The threshold of microsaccade magnitude is determined by the depressed region of synapse strength *S*_*j*_ in space (in Eq. (3)), which strongly depends on the width *σ*_1_ of tuning curve *R*_*j*_. The input to V1 neurons depends more strongly on the depressed region, but weakly on the width *σ*_2_ of tuning curve *W*_*ij*_. The smaller the width *σ*_1_, the smaller the synapse depressed region, leading to the smaller the threshold of microsaccade magnitude.

### “Sharper is better” for microsaccades

**Figure 7:**
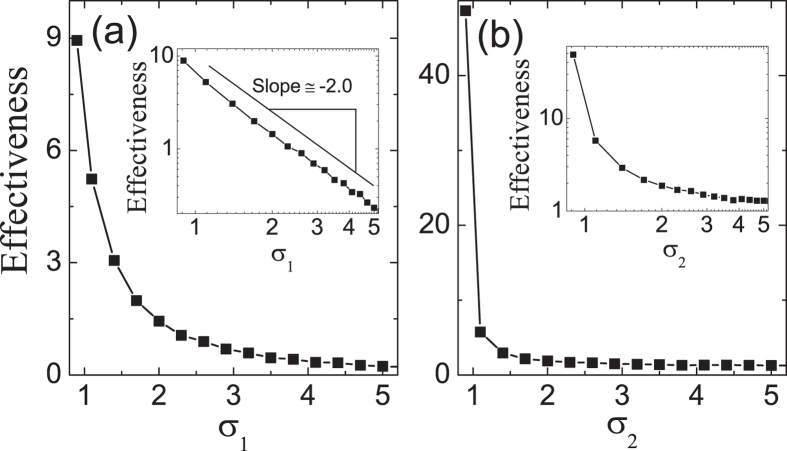
Microsaccade-related effectiveness as a function of the widths *σ*_1_ (**a**) and *σ*_2_ (**b**) of Gaussian tuning curves *G*_1_ and *G*_2_, respectively. The insets in (**a**) show a power-law behavior with an exponent −2 as the increase of *σ*_1_. The inset in (**b**): similar power law does not hold for *σ*_2_. The parameters are 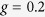, 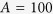, 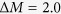, 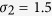 (**a**) and 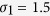 (**b**). Data are averaged over 20 independent runs.

**Figure 8:**
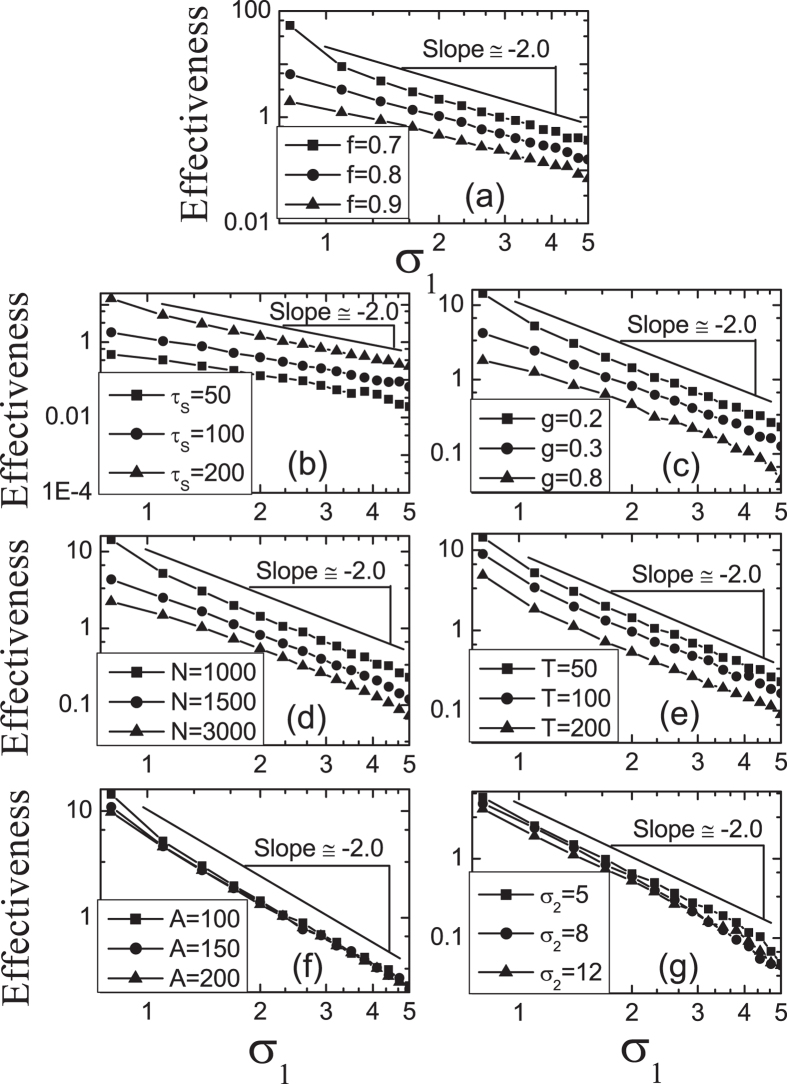
Robust power-law behavior with exponent −2 as the increase of σ1 against some parameters, *f* (**a**), 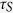 (**b**), *g* (**c**), *N* (**d**), *T* (**e**), *A* (**f**) and *σ*_2_ (**g**). The parameters are 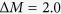 (**a**–**g**), 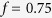 (**b**–**g**), 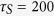 ms (**a**,**c**–**g**), 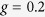 (**a**,**b**,**d**–**g**), 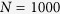 (**a**–**c**,**e**–**g**), 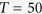 ms (**a**–**d**,**f**,**g**), 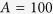 (**a**–**e**,**g**) and 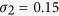 (**a**–**f**). Data are averaged over 20 independent runs.

Bell-shaped tuning curves are widely used to encode variables in the external world by many sensory and cortical neurons. Several studies have indicated that information conveyed by bell-shaped tuning curves increases as they decrease in width^28^,^29^,^30^,^31^. This is the so-called “sharper is better” effect. Especially, for the visual system, this has been experimentally found in many aspects of perceptual learning^29^,^30^. For example, the ability of trained monkeys to discriminate small changes is improved by sharpening of tuning curves in V1 neurons^29^. In our model, the microsaccade-related effectiveness reflects the relative change of neural responses after the onset of microsaccade, which supports the view: our neural system has evolved to optimally detect changes in our environment by moving eyes^6^. So, the effectiveness could be used to denote perceptual function. We focus on the effects of tuning curves *G*_1_ and *G*_2_ on the effectiveness. Clearly, Fig. 7 shows that microsaccade-related neural response displays the property “sharper is better”: the smaller the width (*σ*_1_ or *σ*_2_) of the Gaussian tuning curve (i.e., the sharper the curve), the larger the effectiveness. Particularly, the microsaccade-related effectiveness exhibits a power-law property as a function of *σ*_1_ with an exponent −2 (inset of Fig. 7(a)). A similar power law does not hold for *σ*_2_ (inset of Fig. 7(b)), while the effectiveness also decreases as the increasing of *σ*_2_. This power-law falloff is unusual, as it implies high effectiveness of response to microsaccades for broad range of *σ*_1_, indicating that this decrease is slower than exponential decay. In addition, we find that the exponent −2 is quite robust against some parameters, such as *f*, 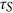, *g*, *N*, *T*, *A*, *σ*_2_ and so on (Fig. 8).

To explore the robust power-law property of effectiveness as a function of *σ*_1_, we give the following approximate calculation. With STD of synapses, it is found that^24^, the total steady-state synaptic conductance resulting from a set of afferents firing at rate *σ*_2_ is proportional to *R*_*j*_*S*_*j*_. So, in the network described by Eq. (1), the V1 response baseline is proportional to the sum 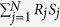 of synaptical inputs from LGN before microsaccade and the V1 response peak is proportional to the sum 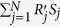 after microsaccade (if we make the approximation that synapses add linearly), where 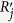 denotes the firing rate in LGN after a microsaccade with small magnitude ΔM. We let the small ΔM not to exceed the synaptic depressed region. By using mean field theory and using the steady synapses in Eq. (3), we can get,


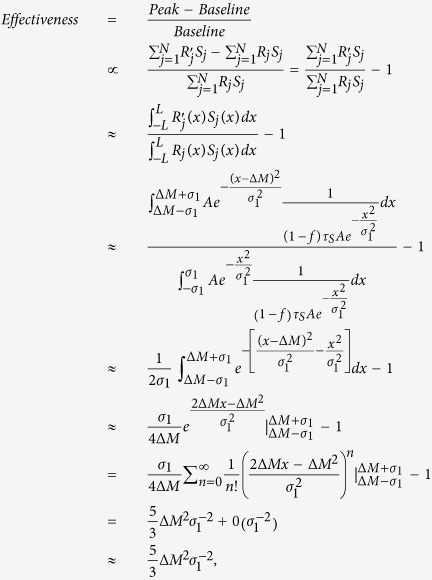


where 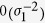 denotes higher order infinitesimal of 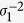 for large *σ*_1_. In Fig. 7(a) and Fig. 8, the power-law relations are given for large *σ*_1_ > 1. In the regime of large *σ*_1_, we here focus on the relation. We can approximately get 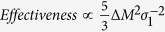. So, the effectiveness can be described by 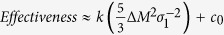, where *k* and *c*_0_ are independent of *σ*_1_ and Δ*M* and are determined by other parameters of V1 neural dynamics in Eqs. (1) and (2). Moreover, the effectiveness is zero for the larger *σ*_1_ because response peak is equal to response baseline in Fig. 4. So, the *c*_0_ is identical to 0. We finally get,


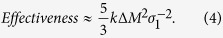


**Figure 9:**
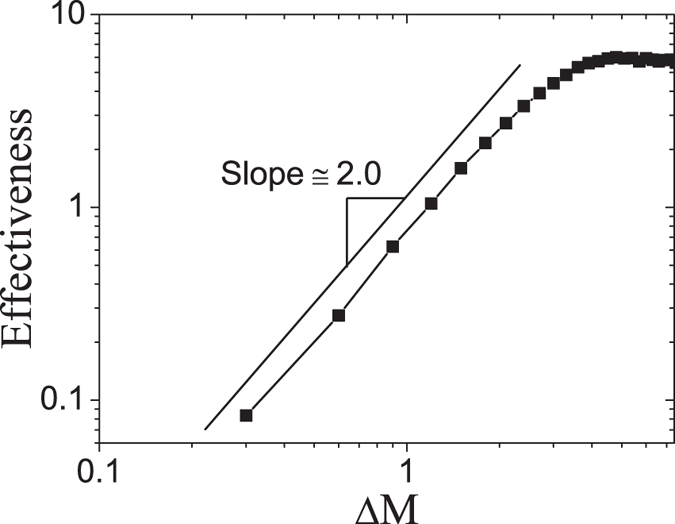
Power-law property with exponent 2 as the increase of ΔΜ. The parameters are given by 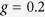, 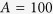, 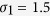 and 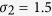. Data are averaged over 20 independent runs.

Obviously, the effectiveness exhibits an approximate power-law form as a function of *σ*_1_ with a robust exponent −2 irrespective of any parameters, such as *f*, 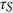, *g*, *N*, *T*, *A* and *σ*_2_, which is consistent with our simulations in Fig. 8. It is noted that, in the derivation of Eq. (4), the microsaccadic size Δ*M* is assumed to be small enough that the microsaccade does not exceed the synaptic depressed region. The effectiveness exhibits another power-law property as a function of small Δ*M* with a positive exponent 2 in Eq. (4). In our simulations, the property is verified for the microsaccade magnitudes smaller than the threshold value producing responsive saturation, shown in Fig. 9. The effectiveness maintains constant for further increasing of the large microsaccade magnitude Δ*M* (shown in the right side of Fig. 9) because of the responsive saturation (see Fig. 6).

## Discussion

By using our model, we extensively study and predict the dependance of microsaccade-related neural responses and responsive saturation value on several key parameters *A*, *σ*_1_ and *σ*_2_, which could be tuned in experiments. For possible verification of our theoretical predictions based on the model, we propose the following feasible experimental designs. The amplitude *A* and width *σ*_1_ of Gaussian tuning curve *G*_1_ evoked by fixated dot can be modified by changing brightness and size of the dot, which can be easily implemented in experiments. Specifically, the more brightness corresponds to the larger amplitude *A*, and larger dot corresponds to larger width *σ*_1_. In addition, the width *σ*_2_ of Gaussian orientation tuning curve *G*_2_ of thalamocortical connecting weights may be modified by training certain microsaccade-related ability, motivated by ref. 29 where the orientation tuning curve at trained orientation became sharper for trained monkey, while modifications of tuning curve were not observed for the monkey which had not been trained. Then results similar to our simulations could be expected.

Particularly, the change of effectiveness due to change of *σ*_1_ displays strong robustness against other parameters with power-law relation. It is plausible that this property reinforces the idea that microsaccades contribute to the vision-detection function: the same microsaccade exhibits different effectiveness for different distribution width *σ*_1_ of light evoked by fixation dot, which seems to support the view: our neural system has evolved to optimally detect changes in our environment by moving eyes^6^.

In conclusion, by using a feedforward network model with STD, several new features of microsaccade-related neural responses are theoretically predicted, which could be tested in experiments. Particularly, we provide a significant prediction “sharper is better”, which has been extensively found in many aspects of perceptual learning. By using mean field theory, we give analytical study on the robust power-law property for “sharper is better”. This prediction, if experimentally verified, would strongly suggest STD in thalamocortical synapses as an important contributor to “sharper is better”. Generally, our study is the first to theoretically predict microsaccade-related neural responses by using biologically plausible model, which could be further tested in experiments. These predictions can give guidance for further experimental studies of the role of microsaccades in visual information processing. These modelling results suggest that, the depression model may be very useful for further investigating behavioral properties and functional roles of microsaccades.

## Additional Information

**How to cite this article**: Zhou, J.-F. *et al.* Model predictions of features in microsaccade-related neural responses in a feedforward network with short-term synaptic depression. *Sci. Rep.* **6**, 20888; doi: 10.1038/srep20888 (2016).
